# Engineering Mycorrhizal Symbioses to Alter Plant Metabolism and Improve Crop Health

**DOI:** 10.3389/fmicb.2017.01403

**Published:** 2017-07-21

**Authors:** Katherine E. French

**Affiliations:** Department of Plant Sciences, University of Oxford Oxford, United Kingdom

**Keywords:** fungal diversity, endosymbiosis, agriculture, synthetic biology, microbial-plant communication, bioprotectants

## Abstract

Creating sustainable bioeconomies for the 21st century relies on optimizing the use of biological resources to improve agricultural productivity and create new products. Arbuscular mycorrhizae (phylum Glomeromycota) form symbiotic relationships with over 80% of vascular plants. In return for carbon, these fungi improve plant health and tolerance to environmental stress. This symbiosis is over 400 million years old and there are currently over 200 known arbuscular mycorrhizae, with dozens of new species described annually. Metagenomic sequencing of native soil communities, from species-rich meadows to mangroves, suggests biologically diverse habitats support a variety of mycorrhizal species with potential agricultural, medical, and biotechnological applications. This review looks at the effect of mycorrhizae on plant metabolism and how we can harness this symbiosis to improve crop health. I will first describe the mechanisms that underlie this symbiosis and what physiological, metabolic, and environmental factors trigger these plant-fungal relationships. These include mycorrhizal manipulation of host genetic expression, host mitochondrial and plastid proliferation, and increased production of terpenoids and jasmonic acid by the host plant. I will then discuss the effects of mycorrhizae on plant root and foliar secondary metabolism. I subsequently outline how mycorrhizae induce three key benefits in crops: defense against pathogen and herbivore attack, drought resistance, and heavy metal tolerance. I conclude with an overview of current efforts to harness mycorrhizal diversity to improve crop health through customized inoculum. I argue future research should embrace synthetic biology to create mycorrhizal chasses with improved symbiotic abilities and potentially novel functions to improve plant health. As the effects of climate change and anthropogenic disturbance increase, the global diversity of arbuscular mycorrhizal fungi should be monitored and protected to ensure this important agricultural and biotechnological resource for the future.

## Introduction

Mutualistic symbiosis is the reciprocally beneficial relationship between two organisms and can have a profound effect on organism fitness, ecology, and evolution (Wade, 2007; Gilbert et al., 2015; Kiers and West, 2015). Symbioses abound across all forms of marine and terrestrial life, from whales (Cetacea) and barnacles (*Coronula diadema*) to fig wasps (*Courtella wardi*) and fig trees (*Ficus carica*) (Jousselin et al., 2003; Nogata and Matsumura, 2006). Some symbioses can also form among three partners, as seen in the exchange of nutrients among three-toed sloths (*Bradypus* spp.), pyralid moths (*Cryptoses* spp.), and algae (*Trichophilus* spp.) (Pauli et al., 2014). However, some of the most intriguing forms of symbioses happen in the microbial world (Keeling and Palmer, 2008). Bacteria and fungi are capable of invading host organisms, altering genetic and metabolic processes along the way (McFall-Ngai et al., 2013). To date, bacterial symbioses have received the most attention due to their complex relationships across many species across all trophic levels, from algae to plants and humans (McFall-Ngai and Ruby, 1991). Fungal symbioses have received less attention despite their key role in terrestrial nutrient exchange and recycling systems (Gadd, 2006; Emery et al., 2015). For example, some fungi (*Neocallimastix* spp.) colonize the guts of cows, helping them digest plant matter (Davies et al., 1993). Other species (ascomycete and basidiomycetes) form symbiotic relationships with cyanobacteria to form lichen, a composite organism (holobiont) that degrades organic and inorganic materials and serve as an important food-source for herbivores (Spribille et al., 2016).

Microbial symbioses have important ecological roles and can also be manipulated to increase the sustainability and resilience of global agricultural systems. To date, the agricultural use of naturally occurring symbioses has focused on promoting symbiosis between nitrogen-fixing bacteria (rhizobia) and legume plants to decrease inorganic nitrogen application on arable fields, which can lead to eutrophication and decline of soil microbial diversity over time (Matson et al., 1997). Arbuscular mycorrhizal fungi (AMF) form an equally important symbiosis with plants that is currently underexploited. This symbiosis is over 400 million years old and predates the symbiotic relationship between nitrogen-fixing bacteria and legumes. In the phylum Glomeromycota there are currently 200 known species from 10 families and many more are likely to be discovered in terrestrial landscapes rich in plant diversity and/or in extreme environments (Bever et al., 2001; Öpik et al., 2010; Ohsowski et al., 2012). In exchange for carbon, mycorrhizae provide plants with Phosphorus (P), minerals, and other nutrients to over 80% of vascular plants (Parniske, 2008). Major crops such as wheat and maize form symbioses with mycorrhizal fungi. The only crops that do not form this symbiosis are from the Brassicaceae and Papaveraceae families (Fester and Sawers, 2011). A growing body of greenhouse and field-scale experiments has shown the positive effect of mycorrhizal inoculation on crop productivity and resilience to environmental stress. In this review, I will provide an overview of our current knowledge of how mycorrhizal symbiosis effects plant metabolism and crop health and provide new insight into how synthetic biology could revolutionize how we harness this symbiosis in the future.

## Mycorrhizal Infection and Host Metabolic Response

**FIGURE 1:**
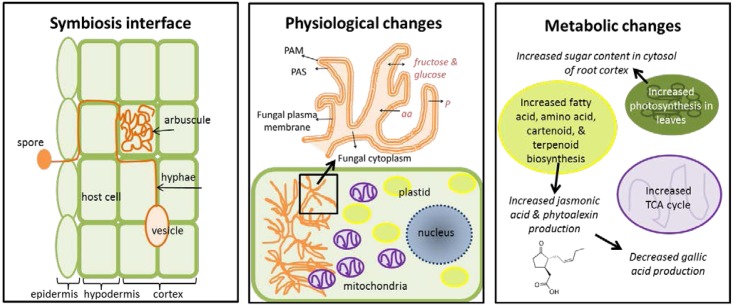
Overview of physiological and metabolic changes induced by mycorrhizal symbiosis with host plants. PAM, plant-derived periarbuscular membrane; PAS, the periarbuscular space; aa, amino acids; P, phosphorus.

Arbuscular mycorrhizal symbiosis dramatically alters plant primary and secondary metabolism in affected roots (**Figure 1**). Upon infection, cells with arbuscules gradually emit enzymes that degrade or stop the suppression of plant cell wall materials (e.g., lignin) and suppress salicylic acid production (which decreases AMF symbiosis) (Smith and Gianinazzi-Pearson, 1988). A shared, permeable membrane is created between the arbuscule and host plant which allows for the exchange of nutrients. This membrane is comprised of three layers: the plant-derived periarbuscular membrane (PAM), the periarbuscular space (PAS) composed of plant and fungal-derived elements, and the fungal plasma membrane (Parniske, 2008). It contains enzymes capable of generating energy gradients for active, bi-directional transport of nutrients and compounds (Smith and Gianinazzi-Pearson, 1988). Infection causes specific physiological changes in host cells. The number of mitochondria increase threefold and migrate toward the arbuscule, the nucleus increases in size, and nuclear chromatin decondenses (allowing for increased transcriptional activity) (Gianinazzi-Pearson, 1996; Lohse et al., 2005). Plastids also increase in number and stromules become more abundant; they can move toward arbuscules, forming a net-like structure over the fungus (Buee et al., 2000; Lohse et al., 2005).

These physiological changes trigger metabolic changes in root cortex cells. Increased numbers of mitochondria and plastids lead to increased energy production (from the TCA cycle) and production of plastid metabolites (fatty acids, amino acids, carotenoids, and terpenoids) respectively (Lohse et al., 2005; Jung et al., 2012). In the cytosol, sugar levels increase due to increased photosynthesis in the above-ground leaves, which favors high efflux rates between the arbuscule and host cell (Smith and Gianinazzi-Pearson, 1988; Berger et al., 2007; Gaude et al., 2015). Levels of jasmonic acid (derived from linoleic acid produced in the plastids) also increase and trigger the production of phytoalexins (defensive compounds). Most of these defensive compounds are nitrogen-rich alkaloids produced by plastids. As these metabolic changes occur, phosphorus is transferred from the mycorrhiza to the host cell in exchange for fatty acids, amino acids, and sugars (fructose and glucose) (Smith and Gianinazzi-Pearson, 1988). The production of anti-fungal compounds (e.g., gallic acid) by the host plant decreases (Gaude et al., 2015).

Foliar secondary metabolism also changes dramatically. Defensive compounds in foliar tissues increase. These compounds include rutin, *p*-hydroxybenzoic acid, antioxidants (flavonoids), and terpenoids (Copetta et al., 2006; Toussaint, 2007; Geneva et al., 2010; Zubek et al., 2015; Kapoor et al., 2016). Earlier studies suggested plant secondary metabolism increased due to increased access to nutrients (specifically, P) provided by the mycorrhizal-endosymbiont (Gupta et al., 2002; Smith et al., 2003). However, several recent experimental studies suggest that increased production of specific secondary compounds does not correlate with increased plant P content (Copetta et al., 2006; Toussaint, 2007). Instead, hormonal changes induced by AMF infection may trigger these metabolic changes. In addition, changes in root chemistry may also lead to the storage of these compounds in foliar tissues. For example, the increased production of glandular trichomes in *Ocimum basilicum* L. when inoculated by *Gigaspora rosea* as noted by Copetta et al. (2006) could be due to increased production of chloroplasts in infected roots. Potentially, sequestering additional volatiles in leaves protects the mycorrhizae living within the hosts’ roots from these bioactive compounds. Additionally, plants may circulate these compounds to where they are most needed.

Arbuscular mycorrhizal fungi symbiosis causes both global (species-independent) and local (species-specific) changes in plant metabolism. Global changes include increased production of amino acids (glutamic, aspartic, and asparagine acid); fatty acids (palmitic and oleic); secondary metabolites (phenyl alcohols, a linolenic acid, apocarotenoids, isoflavonoids); plant hormones (oxylipin, cytokinins, and jasmonic acid); activation of the oxylipin pathway; and increased sugar metabolism (Fernández et al., 2014; Gaude et al., 2015; Rivero et al., 2015). In contrast, levels of specific secondary compounds increase according to plant species identity. For example, Schweiger et al. (2014) found that inoculating *Plantago lanceolata*, *P. major*, *Veronica chamaedrys*, *Medicago truncatula*, and *Poa annua* with *Rhizophagus irregularis* caused 18–45% of each species core metabolomes and increased species-specific compounds (e.g., sorbitol in *P. lanceolata*). Different mycorrhizal fungi can also produce different metabolic effects. For example, repeated pot experiments have shown that *Funneliformis mosseae* causes more metabolic changes than *R. irregularis* (Rivero et al., 2015).

## Impact on Crop Health

Arbuscular mycorrhizal fungi symbiosis can boost plant defenses against pathogens. Previous studies have reported mycorrhizal-induced protection against fungal (*Alternaria, Fusarium*, *Phytophthora*, *Pythium*, *Rhizoctonia*, and *Verticillium)*, bacterial (*Ralstonia solanacearum* and *Pseudomonas syringae*), nematode (*Pratylenchus* and *Meloidogyne*), and insect (*Otiorhynchus sulcatus*) damage (Garcia-Garrido and Ocampo, 1989; de la Peña et al., 2006; Fritz et al., 2006; Pozo and Azcón-Aguilar, 2007; Jung et al., 2012). A recent meta-analysis by Veresoglou and Rillig (2012) suggest inoculation of crops with mycorrhiza reduces fungal infections by 30–42% and nematode infestations by 44–57%. This protection results from passive and active activation of plant secondary metabolism by AMF. Passively, AMF infection causes host plants to produce and store highly potent defensive compounds (alkaloids and terpenoids). These are stored in trichomes and vacuoles and can be released at will (Wink, 1993; Champagne and Boutry, 2016). More actively, external and internal fungal hyphae may sense pathogen effectors and other secondary compounds in the surrounding environment (soil and apoplast, respectively) and ‘warn’ host cells by producing lipo-chitooligosaccharides (LCOs) and short chito-oligosaccharides (Cos) (Kosuta et al., 2003; Maillet et al., 2011; Bonfante and Genre, 2015; Zipfel and Oldroyd, 2017). These messages may transmit through the host plant from cell to cell through the plasmodesmata.

Drought tolerance also increases under inoculation with AMF. This may be due to increased production and accumulation of the sugar trehalose in affected plant cells. Trehalose forms a gel-like substance that attaches to cellular compartments and stabilizes lipid bilayers (Müller et al., 1995; Richards et al., 2002; Lunn et al., 2014). During desiccation organelles remain intact and can spring back to life under favorable environmental conditions (Wingler, 2002). For example, Adams et al. (1990) showed that *Selaginella lepidophylla*’s ability to withstand long-term desiccation was due to high levels of trehalose which formed 12.5% of plant body mass. As many high plants do not produce trehalose, this sugar may potentially be supplied by fungal endosymbionts. Trehalose has been detected in the roots of trees and vascular plants inoculated with ectomycorrhizal and arbuscular mycorrhiza (Müller et al., 1995). Increased trehalose production alters plant carbohydrate metabolism by decreasing sugar and starch levels (Wagner et al., 1986). Providing this benefit to host plants thus comes at a cost to mycorrhizae and emphasizes the mutualism of this symbiosis.

Arbuscular mycorrhizal fungi can also increase tolerance to heavy metals in crops. The reasons for this are still unclear. Potentially, the chitin and melanin found in the cell walls of AMF hyphae may bind metals in the surrounding soil and/or in the host plant (Morley and Gadd, 1995; Ruscitti et al., 2011; Eisenman and Casadevall, 2012). Melanin in particular is well-known for its ability to protect fungi from a variety of harsh environmental conditions, including nuclear radiation (Zhdanova et al., 2000). Non-mycorrhizal fungi (primarily *Aspergillus*, *Phanerochaete chrys*, and *Trichoderma*) have been shown to absorb and incorporate into their cell walls up to 90% of metal ions from soil contaminated with cadmium (Mohsenzadeh and Shahrokhi, 2014), silver (Aksu, 2001), uranium (Wang and Zhou, 2005), and lead (Jianlong et al., 2001). Fungi also use chelating proteins (e.g., phytochelatins and metallothioneins) and metabolites (e.g., oxalate) to deactivate the toxicity of metals (Tomsett, 1993; Sayer and Gadd, 1997). This absorption often triggers changes in fungal metabolism and can cause fungi to change color (e.g., orange and black) as new compounds are produced (Baldrian, 2003). In ectomycorrhiza, Blaudez et al. (2000) have shown that metal ions are deposited throughout the cell wall (50%), cytoplasm (30%), and vacuole (20%). This highlights that multiple mechanisms may be used by mycorrhiza to immobilize metal toxins. The hyphae of ectomycorrhizae exposed to heavy metals also seem to proliferate (Darlington and Rauser, 1988) suggesting they may confer some fitness advantage to fungi. Mycorrhizae may also alter host metabolism to respond to metal toxicity. Shabani et al. (2016) have shown that inoculation of *Festuca arundinacea* with *Funneliformis mosseae* increased the transcription of host metallothioneins and ABC transporters (which aid in the excretion of toxins) in nickel-contaminated soil.

## Inoculum, Synthetic Biology and the Development of Fungal Chasses

Over the past 20 years most attempts to harness AMF diversity to improve crop health have focused on increasing fungal diversity by encouraging plant species diversity (in native habitats like grasslands). In addition, a number of initiatives have sought to create AMF inoculum optimized to increase plant growth. This inoculum often comprises of soil taken from (presumably, fungal-rich) habitats and transferred to arable fields. Although the species-composition of native mycorrhizal communities may vary globally depending on local vegetation, elevation, climate, and soil chemistry, most AMF have low host-specificity (Lee et al., 2013; Veresoglou and Rillig, 2014; Peay et al., 2016). Since AMF readily form symbiosis with multiple plant species, mycorrhizae isolated from one location have the potentially to successfully colonize plants at other sites. Stahl et al.’s (1988) research established that native strains of AMF from non-disturbed sagebrush grasslands increased the biomass and tissue phosphorus content of vegetation planted on reclaimed coal pits in Wyoming. Native soil inocula rich in mycorrhizae have increased the productivity and vegetation cover of American prairies (Richter and Stutz, 2002), Belgian species-rich grasslands (Torrez et al., 2016), and land reclaimed from Mercury mining in California (Emam, 2016). Some mycorrhizae have more beneficial traits than others and in the future inoculum containing these species could be developed. For example, three AMF with hyphae 10x longer than usual mycorrhiza are *Acaulospora laevis* (10.55 cm), *Glomus calospora* (12.3 cm), and *Glomus tenue* (14.2 cm) (Smith and Gianinazzi-Pearson, 1988). Longer hyphae could increase fungal phosphorus uptake and transfer to plant hosts, increasing the production of beneficial phytoalexins and plant growth. In addition, metagenomic research on the microbial communities of biodiverse and/or extreme environments suggests there are many other AMF species which could be exploited further. For example, three new species of AMF (*Diversispora omaniana*, *Septoglomus nakheelum*, and *Rhizophagus arabicus* spp. nov.) were identified in 2014 from environmental samples in the Arabian desert and could be propagated as inoculum for crops grown in arid regions in other parts of the world (Symanczik et al., 2014).

**FIGURE 2:**
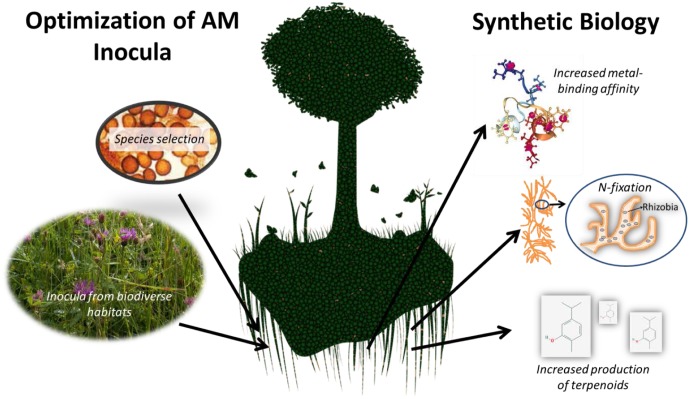
Mycorrhizal symbiosis can be harnessed for agriculture by optimizing soil inocula and through synthetic biology. Image credits (left to right): spores of *Glomus* spp. (IVAM); tree and root image (modified) (CC0 Public Domain); metallothionein from sea urchin (*Strongylocentrotus purpuratus*) (PDB ID 1QJK; Riek et al., 1999); carvacrol (PubChem). All other images belong to the author.

Synthetic biology could draw upon arbuscular mycorrhizal diversity to increase the effect of AMF on plant health (**Figure 2**). Synthetic biology can increase the expression of native host genes by altering transcription rates or by inserting new genes from foreign organisms (Khalil and Collins, 2010). In addition, specific traits could be selected for and expressed in modified fungal chasses. To date, synthetic biology initiatives have focused on using filamentous fungi to produce high value compounds (anti-tumor and antibiotics mainly) (Mattern et al., 2015; Xiao and Zhong, 2016). Currently, the biosynthetic pathways of 197 compounds linked to 779 nucleotide records from 174 fugal species are known and can be accessed via a public database^1^ (Li et al., 2016). The majority of these genes (98%) come from the Ascomycota family and there is a strong bias toward *Aspergillus*.

Currently, there is little to no research on the use of mycorrhizae in synthetic biology, either to improve crop health or to perform advanced biological functions (e.g., mycoremediation). To initiate this process *R. irregularis* (formerly *Glomus intraradices*) could serve as an initial chassis because it is the only mycorrhizae with a fully sequenced genome (Franz Lang and Hijri, 2009; Tisserant et al., 2013). This extensive knowledge of this mycorrhizal host genome could overcome the challenges bioengineering fungi face including (1) the location of biosynthetic gene clusters (BGCs) on multiple loci and (2) the control of BGCs by shared *cis*-regulatory elements (van der Lee and Medema, 2016). Promoters and other regulators of gene expression (e.g., transcription factors) currently developed for use in *Aspergillus niger* and *Penicillium chrysogenum* could be trialed out in these mycorrhizae (Polli et al., 2016; Wanka et al., 2016). Potential genetic targets in *R. irregularis* for manipulation are listed in **Table 1**.

**Table 1:** Genetic targets in *Rhizophagus irregularis*.

Potential Use	UniProt Entry	Protein name	Gene name	Length	Mass (Da)
Drought tolerance	A4QMP6	Trehalase (EC 3.2.1.28) (Alpha-trehalose glucohydrolase)	NTH1	740	86,029
Drought tolerance	A4QMP8	Trehalose-6-phosphatase (Fragment)	TPS2	179	19,765
Heavy metal tolerance	B0AZW1	Metallothionein 1	ntMT1	71	7,202
Nitrogen uptake	D7P896	Nitrate transporter (Fragment)		329	35,823
Nutrient exchange	C8YXI2	Aquaporin 1	AQP1	253	27,190
Phosphorus uptake	G0Z6L2	Phosphate transporter (Fragment)	PT	81	8,573
Phosphorus uptake	Q8X1F6	Phosphate transporter		521	58,478
Plant defense	Q9C0Q8	Chitin synthase (EC 2.4.1.16) (Fragment)	CHS	205	23,115
Symbiosis	B5U322	Germinating spore putative ATP-sulfurylase (Fragment)		82	9,191
Symbiosis	C7EXJ7	Elongation factor 1-alpha (Fragment)	EF1-alpha	255	27,925
Symbiosis	Q9UV76	MYC2 (Fragment)	myc2	286	33,156
Symbiosis	Q2V9G7	Elongation factor 1-alpha (Fragment)	EF1-alpha	306	33,228
Symbiosis	Q659Q9	Elongation factor 1-alpha (Fragment)	tef1a	110	12,346
Symbiosis	Q9UV77	MYC1 (Fragment)	myc1	370	41,872

*The table lists the potential agricultural use of the gene, the UniProt entry, gene name, and protein name, length, and mass*.

Potential applications of synthetically modified mycorrhizae include increased phosphorus uptake, increased production of economically valuable terpenoids (e.g., antibiotic monoterpenes such as carvacrol) in host plants, and potentially even nitrogen-fixation. The latter could be achieved by modifying AMF metabolism or by engineering N-fixing bacteria to engage in symbiosis with specific AMF strains (Manchanda and Garg, 2007). *Burkholderia* spp. engage with *Gigaspora* and *Scutellospora* with as many as 250,000 bacteria per spore (Bianciotto et al., 2000; Artursson et al., 2006), indicating that mycorrhiza have a natural capacity to engage in symbiosis with bacteria. In addition, introducing metallothioneins from other fungi, bacteria, or even higher eukaryotes such as sea urchins into mycorrhiza could improve their ability to protect host plants from metal-contaminated soils. As mycorrhizae do not sexually reproduce, there is little chance these genetic changes would enter into native mycorrhizal gene pools (Pawlowska, 2005). These steps could usher in a revolution in the use of mycorrhiza in synthetic biology.

## Conclusion

Arbuscular mycorrhizal symbioses with plants hold immense promise for the development of more sustainable agricultural systems (Gosling et al., 2006; Garg and Chandel, 2010). Fungi are already extensively used in biotechnology to produce antibiotics, anti-cancer drugs, pigments, bioethanol, and biomaterials (Bennett, 1998; Adrio and Demain, 2003; Cragg et al., 2015; Haneef et al., 2017). To date, arbuscular mycorrhizae have received less attention, despite their dramatic effect on plant metabolism and host resilience to environmental stresses. This fusion of plant and fungal endophyte increases the production of specific plant secondary metabolism products (e.g., fatty acids and terpenoids) and redirects the products of plant primary metabolism (e.g., fructose and glucose) to the fungal partner. This symbiosis also appears to increase the tolerance of crops to pathogen, water, and heavy metal stresses through a variety of mechanisms. Advances in metagenomic sequencing will allow us to promote native AMF diversity while boosting crop fitness (Lumini et al., 2011). The tools and techniques provided by synthetic biology may also lead to new innovations in how these symbioses function and the benefits provided to host plants. Future research should focus on identifying key mycorrhizal genes that affect plant growth and begin experimenting with genetic modification of potential chasses AMF, specifically *R. irregularis*. Rising climate change and anthropogenic disturbance of native ecosystems may harm the diversity and functioning of AMF across the world (Fitter et al., 2000; Antoninka et al., 2009; Classen et al., 2015; French et al., 2017). Conservation efforts must now extend below the soil if we are to ensure the preservation of this resource for the future (Bodelier, 2011).

## Author Contributions

KF conceived and wrote the manuscript.

## Conflict of Interest Statement

The author declares that the research was conducted in the absence of any commercial or financial relationships that could be construed as a potential conflict of interest.

## Footnotes

1 http://mibig.secondarymetabolites.org/
